# Impact of sequential (first- to third-generation) EGFR-TKI treatment on corrected QT interval in NSCLC patients

**DOI:** 10.3389/fonc.2024.1330165

**Published:** 2024-05-07

**Authors:** Tian Gan, Jindong Chen, Hao Wang, Conghui Shang, Siqi Xi, Zixu Fan, Ben He, Min Zhang, Liang Zhao

**Affiliations:** Department of Cardiology, Shanghai Chest Hospital, School of Medicine, Shanghai Jiao Tong University, Shanghai, China

**Keywords:** non-small cell lung cancer, epidermal growth factor receptor-tyrosine kinase inhibitor, corrected QT interval, risk factor, advanced lung cancer

## Abstract

**Objective:**

To evaluate the impact of sequential (first- to third-generation) epidermal growth factor receptor tyrosine kinase inhibitor (EGFR-TKI) treatment on top-corrected QT interval (top-QTc) in non-small cell lung cancer (NSCLC) patients.

**Methods:**

We retrospectively reviewed the medical records of NSCLC patients undergoing sequential EGFR-TKI treatment at Shanghai Chest Hospital between October 2016 and August 2021. The heart rate (HR), top-QT interval, and top-QTc of their ECGs were extracted from the institutional database and analyzed. Logistic regression was performed to identify predictors for top-QTc prolongation.

**Results:**

Overall, 228 patients were enrolled. Compared with baseline (median, 368 ms, same below), both first-generation (376 ms vs. 368 ms, *p* < 0.001) and sequential third-generation EGFR-TKIs (376 ms vs. 368 ms, *p* = 0.002) prolonged top-QT interval to a similar extent (*p* = 0.635). Top-QTc (438 ms vs. 423 ms, *p* < 0.001) and HR (81 bpm vs.79 bpm, *p* = 0.008) increased after first-generation EGFR-TKI treatment. Further top-QTc prolongation (453 ms vs. 438 ms, *p* < 0.001) and HR increase (88 bpm vs. 81 bpm, *p* < 0.001) occurred after treatment advanced. Notably, as HR elevated during treatment, top-QT interval paradoxically increased rather than decreased, and the top-QTc increased rather than slightly fluctuated. Moreover, such phenomena were more significant after treatment advanced. After adjusting for confounding factors, pericardial effusion and lower serum potassium levels were independent predictors of additional QTc prolongation during sequential third-generation EGFR-TKI treatment.

**Conclusion:**

First-generation EGFR-TKI could prolong top-QTc, and sequential third-generation EGFR-TKI induced further prolongation. Top-QT interval paradoxically increased and top-QTc significantly increased as HR elevated, which was more significant after sequential EGFR-TKI treatment. Pericardial effusion and lower serum potassium levels were independent predictors of additional QTc prolongation after sequential EGFR-TKI treatment.

## 1 Introduction

Lung cancer is one leading cause of death, of which non-small cell lung cancer (NSCLC) accounted for approximately 85% of cases (1). Thanks to the discoveries of oncogenic driver mutations, the treatment of NSCLC has been revolutionized by molecularly targeted therapies (2), of which tyrosine kinase inhibitor (TKI) targeting epidermal growth factor receptor (EGFR) has been widely prescribed for NSCLC patients (3), especially in Asian patients whose EGFR mutation prevalence reached 35%–50% (4). Despite the superiority of the first-generation EGFR-TKIs over chemotherapy in improving patients’ survival, such as gefitinib and erlotinib (5), acquired tolerance still occurs in over half of NSCLC patients (6). Also, osimertinib, a third-generation EGFR-TKI, has shown better efficacy than first-generation EGFR-TKIs with much less tolerance (7).

Despite the excellent antitumor effect, EGFR-TKIs could increase the risk of QT interval prolongation, which facilitates ventricular arrhythmia, possibly due to the interaction with potassium channel proteins of cardiomyocytes (8). The EGFR-TKIs share similar pharmacological mechanisms, yet the third-generation EGFR-TKI exerts its function via irreversible binding to the EGFR tyrosine kinase, which is distinguished from the reversible binding capacity of the first-generation EGFR-TKI (9). It remains unclear if stronger binding to EGFR tyrosine kinase could cause more significant QT interval prolongation. Moreover, due to the acquired tolerance of first-generation EGFR-TKI, many NSCLC patients need to take third-generation EGFR-TKI instead, and the impact of such sequential medication on QT interval is also unelucidated.

Theoretically, third-generation EGFR-TKI has a less off-target effect and therefore should induce less cardiotoxicity. However, it remains unclear if different EGFR-TKIs exert a similar impact on QTc, even taking the higher selectivity of third-generation EGFR-TKI into consideration. Moreover, although sequential (first- to third-generation) EGFR-TKI treatment has been frequently used in clinical practice, its impact on QTc remains largely unclear. Furthermore, TKI-induced QT prolongation was observed in approximately 20% of patients (8), yet, to the best of our knowledge, the characteristics of these patients remain unclear. Hence, this study is conducted to investigate the impact of first-generation and sequential third-generation EGFR-TKI treatments on QT interval and the risk factors of QT/QTc prolongation in this subgroup of patients.

## 2 Method

### 2.1 Study population

The study population of this single-center retrospective observational study was selected from the institutional database. The present study was conducted in conformity with the Declaration of Helsinki (as revised in 2013). The institutional ethics committee approved the study protocol and waived written consent.

The medical records of 7,188 NSCLC patients undergoing targeted therapy between October 2016 and August 2021 were reviewed, and a consecutive cohort of 228 patients undergoing sequential first- and third-generation EGFR-TKIs was selected. To avoid confusion about sequential first- and third-generation EGFR-TKI treatments with third-generation EGFR-TKI treatment alone, which was absent in our study, any data obtained after initiation of third-generation EGFR-TKI treatment were documented as during sequential third-generation EGFR-TKI treatment.

The inclusion criteria are as follows: (1) The patients were diagnosed with stages III–IV NSCLC according to World Health Organization (WHO)’s histological classification and the UICC/AJCC TNM classification; (2) EGFR mutation was detected via molecular pathology; (3) sequential third-generation EGFR-TKI was prescribed due to acquired tolerance of first-generation EGFR-TKI, either as monotherapy or combined with chemotherapy; (4) electrocardiogram (ECG) data were available before and during medication; and (5) patients with a score of 0–2 assessed by Eastern Cooperative Oncology Group’s performance status (ECOG PS).

Exclusion criteria included: (1) age < 18; (2) baseline corrected QT interval (QTc) ≥ 450 ms; (3) cardiac arrhythmias causing unreliable QT interval measurement, including frequent ventricular/atrial contraction, persistent atrial fibrillation, bundle branch heart block, or ventricular pacing; (4) medical history of pacemaker implantation, myocardial infarction, coronary artery bypass grafting, or percutaneous coronary intervention; (5) concurrent medication affecting QT interval, such as amiodarone and sotalol; (6) rheumatic heart disease, hypertrophic/dilated cardiomyopathy; and (7) NYHA classes III–IV.

### 2.2 Acquisition of ECG and QTc

All ECGs of the study population were obtained at diagnosis (before antitumor treatment, baseline), at least 1 month after first- and third-generation EGFR-TKI treatments, considering the 29.3-day median interval between the initiation of EGFR-TKI treatment and QT prolongation (10). ECG files were obtained repeatedly, and the mean heart rate was calculated and documented to minimize emotional influence such as anxiety. The tracing speed of standard 12-lead ECG was set to 25 mm/s paper speed, and an amplitude of 0.1 mV/mm was used. All ECG parameters such as QT interval and heart rate (HR, recorded as beats per minute [bpm]) were obtained by using the hospital Cardiac Science ECG system and manually verified by an experienced cardiologist who was blinded to grouping.

The QT interval was defined as the time interval from QRS onset (Q) to where the isoelectric line intersected with the tangential line drawn at (1) the maximal downslope if the T-wave was positive or (2) at the maximal upslope if the T-wave was negative (T_end_). The QT interval was measured in pericardial lead V5, which reflected the potential from the left ventricular free wall (11). According to Bazett’s method, QTc (QT/RR^1/2^) was calculated to correct for heart rate. Top-QTc refers to the maximal value of QTc among all ECGs of every patient, and top-QT interval refers to the QT interval corresponding to the top-QTc. Any discrepancy between automatic and manual measurements in one ECG was resolved by consulting another senior cardiologist.

### 2.3 Definition and grading criteria of QTc interphase prolongation

The prolongation of top-QTc was classified according to four grades according to The National Cancer Institute Common Terminology of Clinical Adverse Events v5.0, including grade 1 (450 ms < QTc ≤ 480 ms), grade 2 (480 ms < QTc ≤ 500 ms), grade 3 (QTc > 501 ms or > 60 ms increase from baseline), and grade 4 (patients showing signs or symptoms of serious arrhythmia and TdP) (12).

### 2.4 Statistical analysis

Continuous variables were expressed as mean ± standard deviation if normally distributed or median (interquartile range [IQR]) if nonnormally distributed, and independent-samples *t*-test or Mann–Whitney *U* test were used for comparison, respectively. Categorical data were expressed as numbers and percentages, and the Chi-square test was performed for comparison. Paired *t*-tests or Wilcoxon signed-rank tests were used to compare the difference between baseline and postintervention values. Univariate logistic regression was performed to identify risk factors of NOAF, of which those with *p*-values < 0.005 were included in multivariate analysis to identify independent risk factors for NOAF. All statistical analyses were conducted by using SPSS 26.0. A two-sided *p*-value < 0.05 was considered statistically significant.

## 3 Results

### 3.1 Baseline characteristics

**Figure 1:**
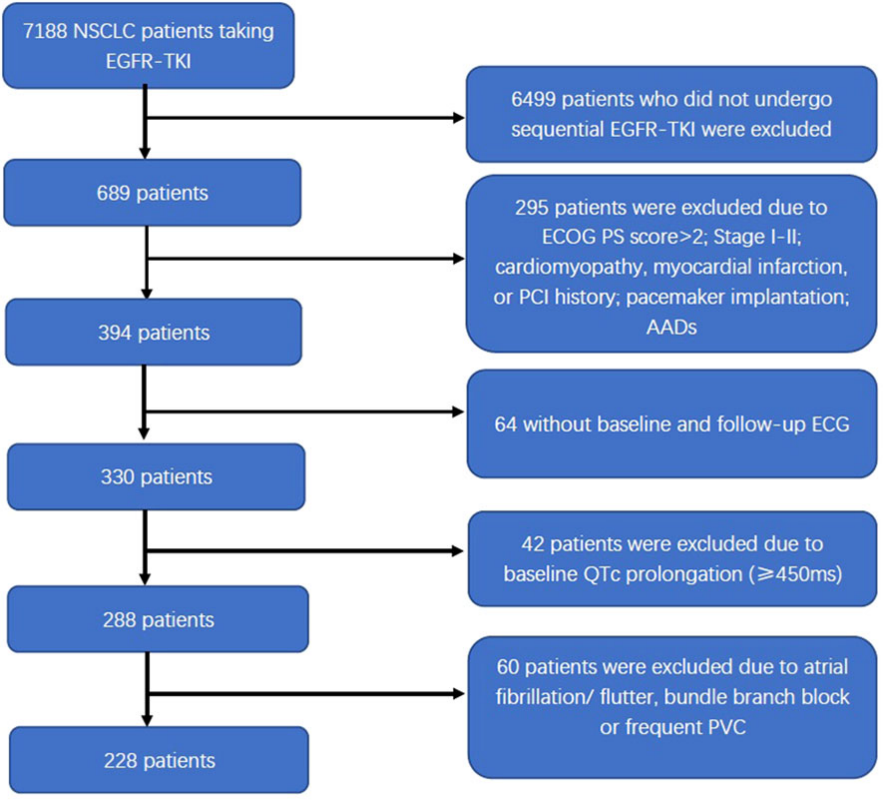
Flow diagram of the patient selection process. NSCLC, non-small cell lung cancer; EGFR-TKI, epidermal growth factor receptor tyrosine kinase inhibitor; ECOG PS, Eastern Cooperative Oncology Group’s performance status; PCI, percutaneous coronary intervention; AADs, antiarrhythmic drugs; ECG, electrocardiogram; QTc, corrected QT interval; PVC, premature ventricular contraction.

Among the 7,188 NSCLC patients undergoing EGFR-TKI treatment, 228 patients diagnosed with advanced NSCLC were included in the study. The detailed selection process is illustrated in **Figure 1**. Overall, the study population included 107 male patients and 121 female patients, with a median age of 60 years old. Of all 228 patients, 214 (93.9%) were diagnosed with stage IV NSCLC, and 194 (85.1%) had peripheral lung cancer. Pericardial effusion was observed in 55 patients. Before taking first-generation EGFR-TKI, exon 19 and 21 mutations were present in 98.2% of patients (*n* = 224). EGFR-TKI monotherapy was administered to 70 patients (70/228, 30.7%), and the other 158 patients (158/228, 69.3%) underwent EGFR-TKI combined with chemotherapy, with the regimen including platinum-paclitaxel in 77 patients (77/228, 33.8%) and platinum-pemetrexed in 81 patients (81/228, 35.5%). The baseline characteristics were summarized in **Table 1**.

**Table 1:** Clinical characteristics of the patients studied.

Clinical characteristics		During first-generation EGFR-TKI treatment			During third-generation EGFR-TKI treatment		
	All patients at baseline	Patients with prolonged top-QTc	Patients with normal top-QTc	P value	Patients with prolonged top-QTc	Patients with normal top-QTc	P value
	(n=228)	(n=75)	(n=153)		(n=128)	(n=100)	
Age (years)	60 (51-66)	58 (49-67)	60 (52-66)	0.606	61 (51-66)	58 (51-66)	0.728
Sex (female, %)	121 (53.1)	48 (64.0)	73 (47.7)	0.021	74 (57.8)	47 (47.0)	0.105
Hypertension (%)	49 (21.5)	14 (18.7)	35 (22.9)	0.467	31 (24.2)	18 (18.0)	0.257
Diabetes (%)	21 (9.2)	5 (6.7)	16 (10.5)	0.352	15 (11.7)	6 (6.0)	0.138
CAD (%)	21 (9.2)	7 (9.3)	14 (9.2)	0.964	13 (10.2)	8 (8.0)	0.576
Smoking (%)	91 (39.9)	26 (34.7)	65 (42.5)	0.257	50 (39.1)	41 (41.0)	0.767
Classification (%)				0.942			0.126
Centric	34 (19.4)	11 (14.7)	23 (15.0)		15 (11.7)	19 (19.0)	
Peripheral	194 (85.1)	64 (85.3)	130 (85.0)		113(88.3)	81 (81.0)	
Pericardial effusion	55 (24.1)	20 (26.7)	35 (22.9)	0.53	38 (29.7)	17 (17.0)	0.026
Laterality (%)				0.594			0.962
Left	103 (45.2)	32 (42.7)	71 (46.4)		58 (45.3)	45 (45.0)	
Right	125 (54.8)	43 (57.3)	82 (53.6)		70 (54.7)	55(55.0)	
EGFR mutation				0.331			0.065
Ex.19 del	135 (59.2)	40 (53.3)	95 (62.1)		69 (53.9)	66 (66.0)	
Non-Ex.19 del	93 (40.8)	35 (46.7)	58 (37.9)		59 (46.1)	34 (34.0)	
L858R	89 (39.0)	33 (44.0)	56 (36.6)		58 (45.3)	31 (31.0)	
Other	4 (1.8)	2 (2.7)	2 (1.3)		1 (0.8)	3 (3.0)	
Cancer stage				0.951			0.938
Stage III	14 (6.1)	4 (5.3)	10 (6.5)		8 (6.3)	6 (6.0)	
Stage IV	214 (93.9)	71 (94.7)	143 (93.5)		120 (93.8)	94 (94.0)	
Brain metastases	14 (6.1)	4 (5.3)	10 (6.5)	0.951	8 (6.3)	6 (6.0)	0.938
Radiotherapy	84 (36.8)	29 (38.7)	55 (35.9)	0.689	49 (38.3)	35 (35.0)	0.61
Surgical history of lung cancer	55 (24.1)	21 (28.0)	34 (22.2)	0.338	29 (22.7)	26 (26.0)	0.558
Platinum-Paclitaxel	77 (33.8)	26 (34.7)	51 (33.3)	0.841	51 (39.8)	26 (26.0)	0.028
Platinum-Pemetrexed	81 (35.6)	26 (34.7)	55 (35.9)	0.849	42 (32.8)	39 (39.0)	0.333
5-HT3 RA	31 (13.6)	11 (14.7)	20 (13.1)	0.741	20 (15.6)	11 (11.0)	0.312
Quinolone	6 (2.6)	3 (4.0)	3 (2.0)	0.643	5 (3.9)	1 (1.0)	0.345
Electrolyte (examined simultaneously with ECG)							
Potassium (mmol/L)	4.0 (3.9-4.3)	4.0 (3.8-4.2)	4.0 (3.8-4.3)	0.245	4.0 (3.8-4.2)	4.1 (3.9-4.3)	0.023
Sodium (mmol/L)	140 (138-141)	139 (138-140)	139 (138-141)	0.399	138 (136-140)	138 (136-140)	0.349
Chlorine (mmol/L)	105 (103-106)	104 (102-106)	105 (103-107)	0.01	103 (99-105)	103 (100-106)	0.254
Calcium (mmol/L)	2.3 (2.3-2.4)	2.3 (2.2-2.4)	2.3 (2.2-2.4)	0.158	2.3 (2.2-2.3)	2.3 (2.2-2.4)	0.615
ECG parameters							
Heart rate (bpm)	79(69-88)	88 (78-99)	78(69-87)	<0.001	91(82-100)	84(74-95)	<0.001
Top-QT (ms)	368 (350-386)	377 (358-402)	374 (354-395)	0.037	381 (361-403)	365 (344-390)	<0.001
Top QTc (ms)	423 (410-435)	458 (453-468)	426 (416-440)	<0.001	464 (456-476)	434 (424-443)	<0.001
Top-QTc prolongation grade (%)							
Grade 0	228 (100.0)	0 (0.0)	153 (100.0)	<0.001	0 (0.0)	100 (100.0)	<0.001
Grade 1	0 (0.0)	67 (89.3)	0 (0.0)		100 (78.1)	0 (0.0)	
Grade 2	0 (0.0)	2 (2.7)	0 (0.0)		10 (7.8)	0 (0.0)	
Grade 3	0 (0.0)	6 (8.0)	0 (0.0)		18 (14.1)	0 (0.0)	
Grade 4	0 (0.0)	0 (0.0)	0 (0.0)		0 (0.0)	0 (0.0)	

Data are presented as median (interquartile range) or n (%). EGFR-TKI, epidermal growth factor receptor tyrosine kinase inhibitor; CAD, coronary artery disease; QTc, corrected QT interval; QT, QT interval; 5-HT3 RA, 5-hydroxytryptamine subtype 3 receptor antagonist.

At baseline, the median QT interval and QTc of the study population were 368 ms and 423 ms, respectively. QTc prolongation occurred in 75 (32.9%) patients after first-generation EGFR-TKI treatment and in 128 (56.1%) patients after sequential third-generation EGFR-TKI treatment. After first-generation EGFR-TKI treatment, QTc prolongation occurrence was more prevalent in female patients (39.7%, 48/121) than male patients (25.2%, 27/107; 39.7% vs. 25.2%, *p* = 0.021); while after sequential third-generation EGFR-TKI treatment, such difference in QTc prolongation became insignificant (female patients, 61.2%, 74/121; male patients, 50.5%, 54/107; 61.2% vs. 50.5%, *p* = 0.105).

### 3.2 Changes of top-QT interval, top-QTc, and HR during EGFR-TKI treatment

Compared with baseline, both first-generation EGFR-TKI and sequential third-generation EGFR-TKI induced a significant increase in top-QTc (median, 438 ms vs. 423 ms, *p* < 0.001; 453 ms vs. 423 ms, *p* < 0.001, first-generation and sequential third-generation EGFR-TKI, respectively, the same below), top-QT interval (median, 376 ms vs. 368 ms, *p* < 0.001; 376 ms vs. 368 ms, *p* = 0.002). HR also increased during first-generation and sequential third-generation EGFR-TKI treatments (median, 81b pm vs. 79 bpm, *p* = 0.008; 88 bpm vs. 79 bpm, *p* < 0.001). After sequential third-generation EGFR-TKI, increased top-QTc (median, 453 ms vs. 438 ms, *p* < 0.001) and HR (median, 88 bpm vs. 81b pm, *p* < 0.001) were observed compared with after first-generation EGFR-TKI, while no significant difference was observed in top-QT interval (median, 376 ms vs. 376 ms, *p* = 0.635).

**Figure 2:**
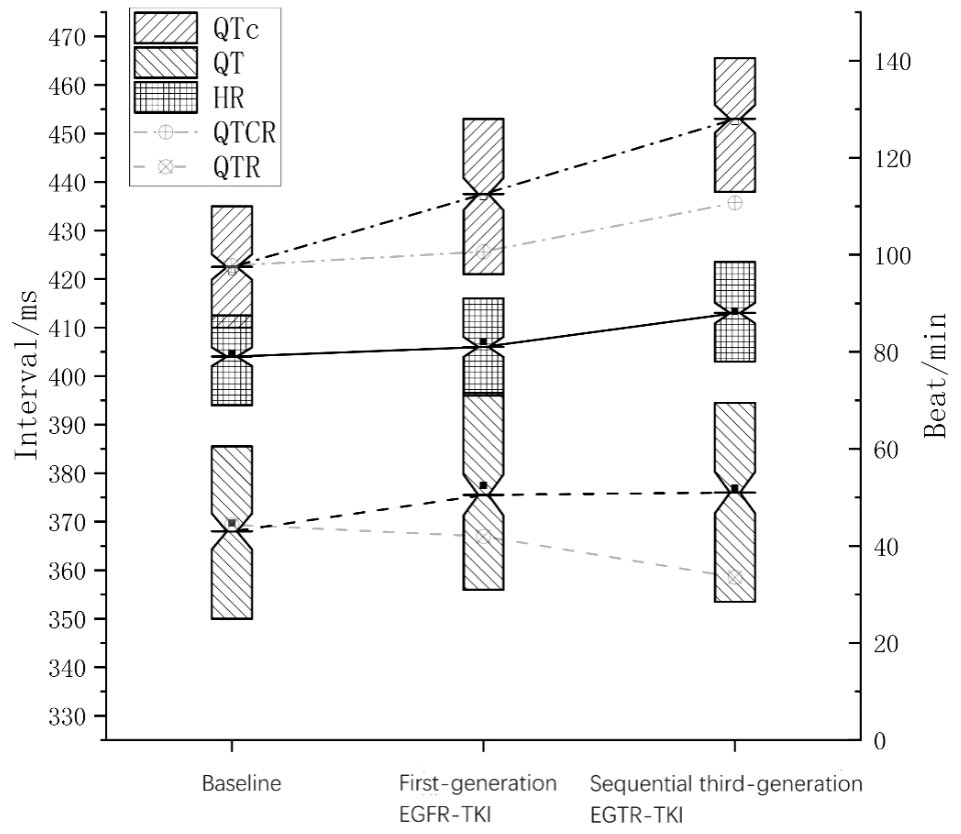
Changes in QT, heart rate, and QTc during sequential EGFR-TKI treatment. The solid line in the middle indicates heart rate; the dashed line, QT interval; the dotted-dashed line, QTc interval. The faded dashed line indicates QT interval at the corresponding heart rate in the general population (QTR), and the faded dotted-dashed line indicates QTc at the corresponding heart rate in the general population (QTCR).

The changes in top-QT interval, top-QTc, and HR during the treatment course are illustrated in **Figure 2**. In the general population, QT interval usually decreases as HR rises, while QTc only exhibits minor fluctuation. It is noteworthy that in the present study, as heart rate elevated during EGFR-TKI treatment, top-QT interval paradoxically increased rather than decreased and top-QTc increased significantly rather than slightly fluctuated, which was more significant after sequential third-generation EGFR-TKI treatment.

### 3.3 Variation of top-QTc prolongation grades induced by different EGFR-TKI treatment

**Figure 3:**
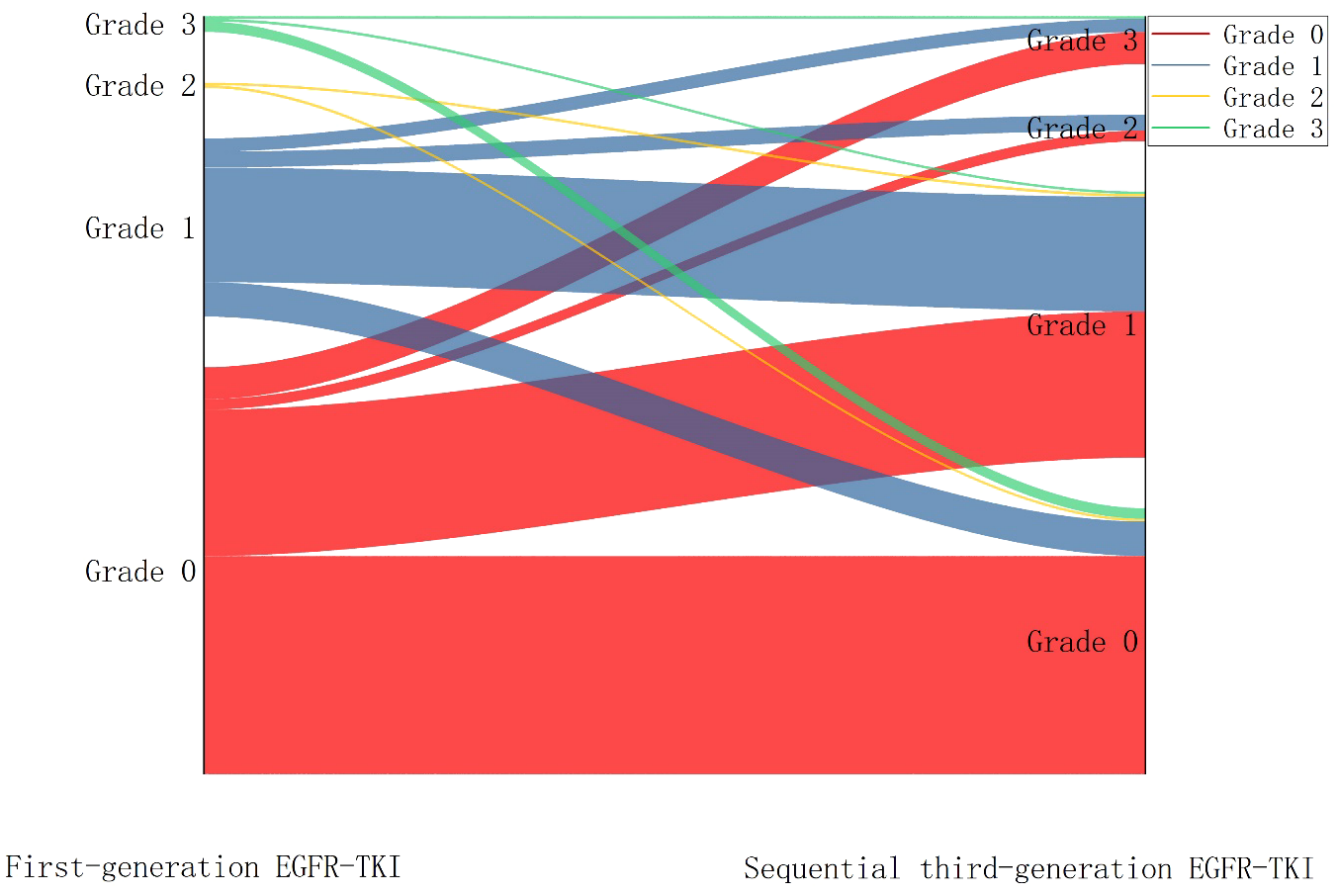
Sankey diagram showing the variation of QTc prolongation after sequential third-generation EGFR-TKI treatment.

Top-QTc prolongation was classified into four grades (0, 1, 2, and 3) based on the prolongation extent. After taking first-generation EGFR-TKI, 153 patients were classified as grade 0 (67.1%), 67 patients as grade 1 (29.4%), and only eight (3.5%) patients as grades 2 and 3. Overall, after sequential third-generation EGFR-TKI treatment, top-QTc prolongation became more prevalent (grades 1–3, *n* = 128). Among the grade 0 patients during first-generation EGFR-TKI treatment (*n* = 153), significant top-QTc prolongation occurred in almost half of the patients (*n* = 71), including grade 1 in 55 patients, grade 2 in four patients, and grade 3 in 12 patients. In the other 75 patients with prolonged top-QTc (grades 1–3) during first-generation EGFR-TKI treatment, the variation of top-QTc prolongation grades seemed diverse without a significant trend. The variation of top-QTc prolongation is illustrated in **Figure 3**.

After sequential third-generation EGFR-TKI treatment, the serum potassium level was below the normal range (3.5–5.5 mmol) in only 12 patients, of which QTc prolongation was absent in four patients and observed in the other eight patients including grade I in six patients, grade II in one patient, and grade III in one patient. Moreover, among the 18 patients with grade III QTc prolongation after sequential third-generation EGFR-TKI treatment, the serum potassium level was within the normal range in 17 patients (range, 3.5–4.8 mmol/L). Notably, the other patient had a slightly lower potassium level (3.4 mmol/L) but the most extended QTc (608 ms).

### 3.4 Risk factors for top-QTc prolongation

**Figure 4:**
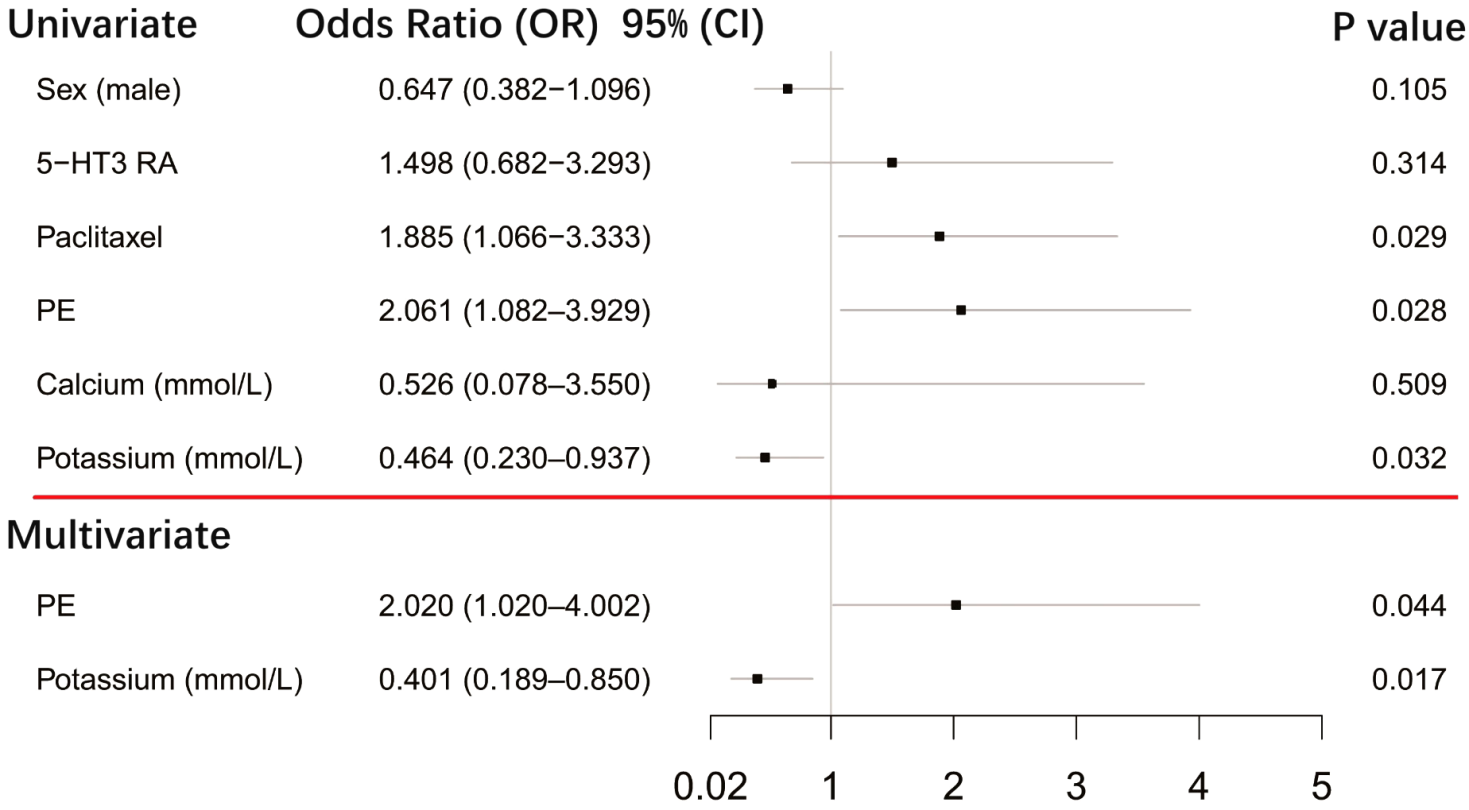
Forest plot showing univariate and multivariate risk factors of top-QTc prolongation after sequential third-generation EGFR-TKI treatment. PE, pericardial effusion; 5-HT3 RA, 5-hydroxytryptamine subtype 3 receptor antagonist.

After first-generation EGFR-TKI treatment, the top-QTc prolongation group had more female patients (*n* = 48) than the control group (*n* = 27) (39.7% vs. 25.2%, *p* = 0.021). Female patients had a tendency of top-QT prolongation (*n* = 74) but did not yield a statistical difference after sequential third-generation EGFR-TKI treatment in the control group (*n* = 54) (61.2% vs. 50.5%, *p* = 0.105). However, as shown in **Figure 4**, after sequential third-generation EGFR-TKI treatment, the univariate analysis yielded that pericardial effusion (*p* = 0.028), paclitaxel history (*p* = 0.029), and lower serum potassium level (*p* = 0.032) were risk factors for additional top-QTc prolongation after sequential third-generation EGFR-TKI treatment. After adjusting confounding factors that could influence QTc, including sex, combined medication of paclitaxel and 5-hydroxytryptamine subtype 3 (5-HT3) receptors antagonist and calcium level, multivariate analysis still yielded pericardial effusion (*p* = 0.044) and lower serum potassium level (*p* = 0.017) as independent risk factors for additional top-QTc prolongation.

## 4 Discussion

The present study focused on EGFR-mutant NSCLC patients who underwent sequential first- to third-generation EGFR-TKI treatment and evaluated the impact of sequential EGFR-TKI treatment on top-QTc. The following is a summary of the main findings: (1) Both first-generation and sequential third-generation EGFR-TKI treatments induced top-QTc prolongation, and sequential third-generation EGFR-TKI induced additional top-QTc prolongation. (2) During the whole EGFR-TKI treatment course, as heart rate elevated, top-QT interval paradoxically increased rather than decreased and top-QTc increased rather than slightly fluctuated, which was more significant after sequential third-generation EGFR-TKI treatment. (3) After adjusting for confounding factors, pericardial effusion and low serum potassium levels were independent risk factors for top-QTc prolongation in patients taking third-generation EGFR-TKI.

As a transmembrane receptor, EGFR is activated via autophosphorylation by tyrosine kinase after being activated by an extra-cellular signal, which subsequently results in the activation of a series of downstream intracellular pathways that promote tumor proliferation and metastasis (13). Small-molecule first-generation EGFR-TKI, such as erlotinib and gefitinib, could reversibly inhibit the autophosphorylation of EGFR and the downstream signaling via binding the catalytic domain (9). Third-generation EGFR-TKI, such as osimertinib, irreversibly inhibit EGFR and therefore is effective for TKI-resistant NSCLC (9).

Previous studies have reported a higher risk of QT prolongation of third- than first-generation EGFR-TKI by comparing patients taking different TKIs (14), which is consistent with our study. Moreover, our study found that in NSCLC patients with normal baseline QTc who underwent first- and third-generation EGFR-TKI sequentially, third-generation EGFR-TKI induced more significant top-QTc prolongation. The major mechanisms included a block of Ether-à-go-go-related gene (hERG) channel, downregulation of phosphoinositide 3-kinase (PI3K), and partial block of KCNQ1/KCNE1 (15–18), which could cause decreased currents of *I*_Kr_, *I*_Ks_, and *I*_CaL_ and could subsequently prolong activation potential duration and QT interval. Moreover, osimertinib and its active metabolite (AZ5104) can also inhibit human epidermal growth factor receptor-2 *in vitro (* 14), which in turn causes downregulation of PI3K and affects membranous sodium channel function (19), therefore inducing further QT interval prolongation.

The association between heart rate and the duration of QT-interval is well recognized that in the general population, QT interval decreases as HR increases (20), and therefore QTc was introduced to evaluate ventricular repolarization regardless of HR. Previous *in vitro* studies have attributed such characteristics to the *I*_Ks_ channel due to its open-state accumulation at a faster heart rate (21, 22). Our study found a longer QT interval even under higher HR after EGFR-TKI treatment, suggesting that TKIs (1) impaired the response of ventricular repolarization to HR variation and (2) caused further prolongation in ventricular repolarization manifested as QT prolongation. The detailed mechanism remains unclear, yet considering the critical role of *I*_Ks_ in regulating action potential duration, it seems possible that third-generation EGFR-TKI may affect the *I*_Ks_ channel to a greater extent.

Pericardial effusion can be frequently observed among advanced NSCLC patients, indicative of a poor outcome (23, 24). Previous studies have observed reduced QRS wave amplitude and T-wave alternan induced by pericardial effusion (25). In our study, we found that pericardial effusion is associated with top-QTc prolongation, with possible mechanisms as follows: (1) In advanced NSCLC patients, pericardial effusion may contain tumor-secreted inflammatory cytokines that could induce inflammation and fibrosis, leading to prolongation of QT interval, such as interleukin (IL)-1, IL-6, IL-8, IL-10, TNF-α, and TGF-β (26, 27). (2) EGFR-TKI could induce pericardial effusion due to cardiac toxicity, such as osimertinib and its metabolite AZ5104, which could induce myocardial injury via inhibiting HER2 (14). These two factors may affect ventricular repolarization and subsequently cause QTc prolongation.

Lower serum potassium is the other independent risk factor for additional top-QTc prolongation after EGFR-TKI alteration. Transmembrane outward potassium current is inhibited at low serum potassium levels, leading to prolonged ventricular repolarization, which is associated with an increased risk of early afterdepolarizations (28). Moreover, hypokalemia could also result in the downregulation of the hERG channel and cause QT prolongation (29), suggesting an increased risk of ventricular arrhythmias. Generally, EGFR-TKI could increase the risk of QTc prolongation, which tends to be aggravated when concomitant low serum potassium level is present (30). In our study, no life-threatening ventricular arrhythmias were observed, and EGFR-TKI-induced QTc prolongation seemed benign. However, during EGFR-TKI treatment, physicians should pay attention to patients’ ECG and electrolyte levels, especially the potassium level.

## 5 Limitation

The present study is a retrospective observational study with a limited sample size, the result of which warrants further verification. Furthermore, this study focused on the impact of sequential first- to third-generation EGFR-TKI treatment on top-QTc in NSCLC patients; further comparison between this subgroup of patients and those who take third-generation EGFR-TKI as initial treatment should be performed. Moreover, NSCLC patients are usually under multiple medications that might affect QTc, such as Paclitaxel, quinolone, and 5-HT3 receptor antagonists. Despite the fact that no significant influence of these medications was observed on QTc in this subgroup of patients, further studies are warranted for verification.

## 6 Conclusion

First-generation EGFR-TKI could prolong top-QTc, and sequential third-generation EGFR-TKI induced further QTc prolongation. As HR elevated, top-QT interval paradoxically increased rather than decreased, and the top-QTc increased rather than slightly fluctuated, which was more significant after sequential third-generation EGFR-TKI treatment. During EGFR-TKI treatment, oncologists should pay close attention to patients’ top-QTc interval variation, especially when combined with pericardial effusion and lower serum potassium level, which were independent risk factors for additional QTc prolongation after sequential EGFR-TKI treatment.

## Data availability statement

The original contributions presented in the study are included in the article/supplementary material. Further inquiries can be directed to the corresponding authors.

## Ethics statement

The studies involving humans were approved by Shanghai chest hospital ethics committee. The studies were conducted in accordance with the local legislation and institutional requirements. Written informed consent for participation was not required from the participants or the participants’ legal guardians/next of kin in accordance with the national legislation and institutional requirements.

## Author contributions

TG: Conceptualization, Writing – original draft. JC: Writing – original draft, Funding acquisition, Software. HW: Data curation, Writing – original draft. CS: Data curation, Writing – original draft. SX: Data curation, Writing – original draft. ZF: Data curation, Writing – original draft. BH: Project administration, Supervision, Writing – original draft. MZ: Project administration, Supervision, Conceptualization, Writing – original draft. LZ: Conceptualization, Project administration, Supervision, Funding acquisition, Writing – review & editing.

## References

[1] Sung H Ferlay J Siegel RL Laversanne M Soerjomataram I Jemal A. Global cancer statistics 2020: GLOBOCAN estimates of incidence and mortality worldwide for 36 cancers in 185 countries. CA Cancer J Clin. (2021) 71:209–49. doi: 10.3322/caac.21660 33538338

[2] Otano I Ucero AC Zugazagoitia J Paz-Ares L. At the crossroads of immunotherapy for oncogene-addicted subsets of NSCLC. Nat Rev Clin Oncol. (2023) 20:143–59. doi: 10.1038/s41571-022-00718-x 36639452

[3] Mok TS Wu Y-L Thongprasert S Yang C-H Chu D-T Saijo N. Gefitinib or carboplatin-paclitaxel in pulmonary adenocarcinoma. New Engl J Med. (2009) 361:947–57. doi: 10.1056/NEJMoa0810699 19692680

[4] Melosky B Kambartel K Häntschel M Bennetts M Nickens DJ Brinkmann J. Worldwide prevalence of epidermal growth factor receptor mutations in non-small cell lung cancer: A meta-analysis. Mol Diagn Ther. (2022), 26(1):7–18. doi: 10.1007/s40291-021-00563-1 PMC8766385 34813053

[5] Hanna N Johnson D Temin S Baker S Brahmer J Ellis PM. Systemic therapy for stage IV non-small-cell lung cancer: american society of clinical oncology clinical practice guideline update. J Clin Oncol. (2017) 35:3484–515. doi: 10.1200/JCO.2017.74.6065 28806116

[6] Oxnard GR Arcila ME Sima CS Riely GJ Chmielecki J Kris MG. Acquired resistance to EGFR tyrosine kinase inhibitors in EGFR-mutant lung cancer: distinct natural history of patients with tumors harboring the T790M mutation. Clin Cancer Res. (2011) 17:1616–22. doi: 10.1158/1078-0432.CCR-10-2692 PMC3060283 21135146

[7] Ramalingam SS Vansteenkiste J Planchard D Cho BC Gray JE Ohe Y. Overall survival with osimertinib in untreated, EGFR-mutated advanced NSCLC. New Engl J Med. (2020) 382:41–50. doi: 10.1056/NEJMoa1913662 31751012

[8] Porta-Sánchez A Gilbert C Spears D Amir E Chan J Nanthakumar K. Incidence, diagnosis, and management of QT prolongation induced by cancer therapies: A systematic review. J Am Heart Assoc. (2017), 6(12):e007724. doi: 10.1161/JAHA.117.007724 PMC5779062 29217664

[9] He J Zhou Z Sun X Yang Z Zheng P Xu S. The new opportunities in medicinal chemistry of fourth-generation EGFR inhibitors to overcome C797S mutation. Eur J Med Chem. (2021) 210:112995. doi: 10.1016/j.ejmech.2020.112995 33243531

[10] Waliany S Zhu H Wakelee H Padda SK Das M Ramchandran K. Pharmacovigilance analysis of cardiac toxicities associated with targeted therapies for metastatic NSCLC. J Thorac Oncol. (2021) 16:2029–39. doi: 10.1016/j.jtho.2021.07.030 34418561

[11] Takenaka K Ai T Shimizu W Kobori A Ninomiya T Otani H. Exercise stress test amplifies genotype-phenotype correlation in the LQT1 and LQT2 forms of the long-QT syndrome. Circulation. (2003) 107:838–44. doi: 10.1161/01.CIR.0000048142.85076.A2 12591753

[12] Kim PY Irizarry-Caro JA Ramesh T Iliescu C Lopez-Mattei JC. How to diagnose and manage QT prolongation in cancer patients. JACC CardioOncol. (2021) 3:145–9. doi: 10.1016/j.jaccao.2021.01.002 PMC8352274 34396315

[13] Ciardiello F Tortora G. EGFR antagonists in cancer treatment. New Engl J Med. (2008) 358:1160–74. doi: 10.1056/NEJMra0707704 18337605

[14] Anand K Ensor J Trachtenberg B Bernicker EH. Osimertinib-induced cardiotoxicity: A retrospective review of the FDA adverse events reporting system (FAERS). JACC CardioOncol. (2019) 1:172–8. doi: 10.1016/j.jaccao.2019.10.006 PMC8352117 34396179

[15] Roden DM. A current understanding of drug-induced QT prolongation and its implications for anticancer therapy. Cardiovasc Res. (2019) 115:895–903. doi: 10.1093/cvr/cvz013 30689740 PMC7967705

[16] Lu Z Wu C-YC Jiang Y-P Ballou LM Clausen C Cohen IS. Suppression of phosphoinositide 3-kinase signaling and alteration of multiple ion currents in drug-induced long QT syndrome. Sci Trans Med. (2012) 4:131ra50. doi: 10.1126/scitranslmed.3003623 PMC3494282 22539774

[17] Jie L-J Li Y-D Zhang H-Q Mao L Xie H-B Zhou F-G. Mechanisms of gefitinib-induced QT prolongation. Eur J Pharmacol. (2021) 910:174441. doi: 10.1016/j.ejphar.2021.174441 34474028

[18] Jin T Hu B Chen S Wang Q Dong X Zhang Y. An in vitro assay of hERG K + Channel potency for a new EGFR inhibitor FHND004. Front Pharmacol. (2018) 9:577. doi: 10.3389/fphar.2018.00577 29904349 PMC5990611

[19] Rasti AR Guimaraes-Young A Datko F Borges VF Aisner DL Shagisultanova E. PIK3CA mutations drive therapeutic resistance in human epidermal growth factor receptor 2-positive breast cancer. JCO Precis Oncol. (2022) 6:e2100370. doi: 10.1200/PO.21.00370 35357905 PMC8984255

[20] Ahnve S Vallin H. Influence of heart rate and inhibition of autonomic tone on the QT interval. Circulation. (1982) 65:435–9. doi: 10.1161/01.CIR.65.3.435 7055864

[21] Romey G Attali B Chouabe C Abitbol I Guillemare E Barhanin J. Molecular mechanism and functional significance of the MinK control of the KvLQT1 channel activity. J Biol Chem. (1997) 272:16713–6. doi: 10.1074/jbc.272.27.16713 9201970

[22] Drici MD Arrighi I Chouabe C Mann JR Lazdunski M Romey G. Involvement of IsK-associated K+ channel in heart rate control of repolarization in a murine engineered model of Jervell and Lange-Nielsen syndrome. Circ Res. (1998) 83:95–102. doi: 10.1161/01.RES.83.1.95 9670922

[23] Imazio M Colopi M and De Ferrari GM. Pericardial diseases in patients with cancer: contemporary prevalence, management and outcomes. Heart (British Cardiac Society). (2020) 106:569–74. doi: 10.1136/heartjnl-2019-315852 31980441

[24] El Haddad D Iliescu C Yusuf SW William WN Khair TH Song J. Outcomes of cancer patients undergoing percutaneous pericardiocentesis for pericardial effusion. J Am Coll Cardiol. (2015) 66:1119–28. doi: 10.1016/j.jacc.2015.06.1332 PMC4560839 26337990

[25] Azarbal A LeWinter MM. Pericardial effusion. Cardiol Clin. (2017) 35:515–24. doi: 10.1016/j.ccl.2017.07.005 29025543

[26] Wu B Sodji QH and Oyelere AK. Inflammation, fibrosis and cancer: mechanisms, therapeutic options and challenges. Cancers (Basel). (2022), 14(3):552. doi: 10.3390/cancers14030552 35158821 PMC8833582

[27] Youssef ME El-Azab MF Abdel-Dayem MA Yahya G Alanazi IS Saber S. Electrocardiographic and histopathological characterizations of diabetic cardiomyopathy in rats. Environ Sci pollut Res Int. (2022) 29:25723–32. doi: 10.1007/s11356-021-17831-6 34845640

[28] Osadchii OE. Mechanisms of hypokalemia-induced ventricular arrhythmogenicity. Fundam Clin Pharmacol. (2010) 24:547–59. doi: 10.1111/j.1472-8206.2010.00835.x 20584206

[29] Mladěnka P Applová L Patočka J Costa VM Remiao F Pourová J. Comprehensive review of cardiovascular toxicity of drugs and related agents. Med Res Rev. (2018) 38:1332–403. doi: 10.1002/med.21476 PMC6033155 29315692

[30] Bian S Tang X Lei W. A case of torsades de pointes induced by the third-generation EGFR-TKI, osimertinib combined with moxifloxacin. BMC Pulm Med. (2020) 20:181. doi: 10.1186/s12890-020-01217-4 32580784 PMC7313192

